# Effects of low-dose rate γ-irradiation combined with simulated microgravity on markers of oxidative stress, DNA methylation potential, and remodeling in the mouse heart

**DOI:** 10.1371/journal.pone.0180594

**Published:** 2017-07-05

**Authors:** John W. Seawright, Yusra Samman, Vijayalakshmi Sridharan, Xiao Wen Mao, Maohua Cao, Preeti Singh, Stepan Melnyk, Igor Koturbash, Gregory A. Nelson, Martin Hauer-Jensen, Marjan Boerma

**Affiliations:** 1 Division of Radiation Health, Department of Pharmaceutical Sciences, University of Arkansas for Medical Sciences, Little Rock, AR, The United States of America; 2 Department of Basic Sciences and Radiation Medicine, Loma Linda University, Loma Linda, CA, The United States of America; 3 Department of Pediatrics, University of Arkansas for Medical Sciences, Little Rock, AR, The United States of America; 4 Department of Environmental and Occupational Health, University of Arkansas for Medical Sciences, Little Rock, AR, The United States of America; Northwestern University Feinberg School of Medicine, UNITED STATES

## Abstract

**Purpose:**

Space travel is associated with an exposure to low-dose rate ionizing radiation and the microgravity environment, both of which may lead to impairments in cardiac function. We used a mouse model to determine short- and long-term cardiac effects to simulated microgravity (hindlimb unloading; HU), continuous low-dose rate γ-irradiation, or a combination of HU and low-dose rate γ-irradiation.

**Methods:**

Cardiac tissue was obtained from female, C57BL/6J mice 7 days, 1 month, 4 months, and 9 months following the completion of a 21 day exposure to HU or a 21 day exposure to low-dose rate γ-irradiation (average dose rate of 0.01 cGy/h to a total of 0.04 Gy), or a 21 day simultaneous exposure to HU and low-dose rate γ-irradiation. Immunoblot analysis, rt-PCR, high-performance liquid chromatography, and histology were used to assess inflammatory cell infiltration, cardiac remodeling, oxidative stress, and the methylation potential of cardiac tissue in 3 to 6 animals per group.

**Results:**

The combination of HU and γ-irradiation demonstrated the strongest increase in reduced to oxidized glutathione ratios 7 days and 1 month after treatment, but a difference was no longer apparent after 9 months. On the other hand, no significant changes in 4-hydroxynonenal adducts was seen in any of the groups, at the measured endpoints. While manganese superoxide dismutase protein levels decreased 9 months after low-dose γ-radiation, no changes were observed in expression of catalase or Nrf2, a transcription factor that determines the expression of several antioxidant enzymes, at the measured endpoints. Inflammatory marker, CD-2 protein content was significantly decreased in all groups 4 months after treatment. No significant differences were observed in α-smooth muscle cell actin protein content, collagen type III protein content or % total collagen.

**Conclusions:**

This study has provided the first and relatively broad analysis of small molecule and protein markers of oxidative stress, T-lymphocyte infiltration, and cardiac remodeling in response to HU with simultaneous exposure to low-dose rate γ-radiation. Results from the late observation time points suggest that the hearts had mostly recovered from these two experimental conditions. However, further research is needed with larger numbers of animals for a more robust statistical power to fully characterize the early and late effects of simulated microgravity combined with exposure to low-dose rate ionizing radiation on the heart.

## Introduction

Exposure to high doses of ionizing radiation may cause cardiac toxicity that involves pericarditis, fibrosis, conduction abnormalities, coronary heart disease and atherosclerosis [1,2]. While an increased risk of cardiovascular disease has long been shown in radiotherapy patients [3–5], adverse cardiovascular effects, such as ischemic heart disease, have also been described following exposure to doses much lower than in radiation therapy, often decades after radiation exposure [6–8]. Astronauts traveling beyond low earth orbit (LEO), as in a mission to Mars or a near earth asteroid, will be continuously exposed to low dose-rate ionizing radiation, including γ-rays released as secondary radiation resultant from particle radiation interacting with the spacecraft or other shielding material [9]. Hence, there is a concern about potential degenerative tissue effects in the heart with the exposure to ionizing radiation that occurs during space travel.

One mechanism by which ionizing radiation may lead to cardiac toxicity is through increased oxidative stress. One characteristic of ionizing radiation is the formation of free radicals, e.g. reactive oxygen species (ROS) [10,11]. An increase in oxidative stress has been observed in Russian cosmonauts and in space flown animal models [12–14]. Recently, Azimzadeh et al., demonstrated a decrease in anti-oxidant proteins, including nuclear factor erythroid 2-related factor 2 (Nrf2) and superoxide dismutase 2 (SOD2), indicative of an increase in oxidative stress in post-mortem left ventricular tissue from Mayak nuclear workers that had been exposed to low dose-rate γ-radiation [15]. Furthermore, Soucy et al. demonstrated increased activity of xanthine oxidase, an enzyme that generates ROS, in the rat aorta following exposure to 5 Gy γ-radiation [16]. Additionally, mitochondria isolated from mice 4 weeks after 2 Gy local heart X-ray irradiation show an increase in ROS levels when presented with an additional insult [17].

Oxidation of glutathione (GSH) to form glutathione disulfide (GSSG) is an important buffering mechanism to reduce ROS levels. Hence, the ratio of tissue levels of GSH to GSSG is used as one indicator of oxidative stress.

Another mechanism by which radiation may affect the heart is through DNA methylation, an important mechanism of maintenance of cellular homeostasis [18,19]. Cardiovascular disease is associated with alterations of DNA methylation [20,21]. Moreover, radiation exposure has recently been shown to alter the DNA methylation patterns in the mouse heart [19]. Methionine and the methionine cycle are critical for the production of S-adenosyl- _L_-methionine (SAM), a major methyl donor [22]. Impairments in the methionine cycle up to 48 hours following radiation have been observed in the breast cancer cell line MDA-MB-361 by O’Leary et al. through the suppression of methionine adenosyltranseferase by the long noncoding RNA, PARTICLE [23]. O’leary et al. observed a transient increase in PARTICLE 4 and 24 hours after 0.25 Gy γ-irradiation and a corresponding decrease in SAM 4 hours after radiation [23]. While methionine metabolism directly influences DNA methylation potential, it also plays an important role in redox status. Increases in oxidative stress result in homocysteine, a reversible product in the methionine cycle, to be diverted away from the methionine cycle; thus, 1) decreasing the ability of the cells or tissues to methylate DNA [24] and 2) supporting the synthesis of the antioxidant GSH through the irreversible transsulfuration of methionine [25]. Importantly, a recent study demonstrated that exposure to low absorbed doses of space radiation resulted in long-term shifts in the heart tissue methionine concentrations, the ratio of SAM to S-adenosylhomocysteine (SAH), and DNA methylation [19].

In addition to space radiation as an occupational hazard, microgravity also presents a unique challenge to an astronaut. Microgravity has been shown to play a role in epigenetics, or DNA methylation, in breast cancer cell lines, human lymphocytes, and lymphoblastoid cells. [26–29]. Furthermore, increased oxidative stress coupled with impairment of antioxidant mechanisms and inflammation have been shown in skeletal muscle following exposure to simulated microgravity [30]. Recently, Mao et al. showed that a combined exposure to hindlimb unloading (HU) and low-dose rate γ-radiation may increase oxidative damage and decrease antioxidant protein content in the mouse brain [31]. To be able to model simultaneous HU and exposure to low-dose rate ionizing radiation, Mao et al. placed cages of hind-limb unloaded mice onto Co-57 plates that emitted ionizing radiation in the form of γ-rays at an average dose rate of 0.01 cGy/h.

Since, the combined effects of low-dose rate radiation exposure and microgravity on the heart are largely unknown, the purpose of the current study was to evaluate indicators of oxidative stress and tissue remodeling in cardiac tissue obtained from the mice in the study of Mao et al. [31].

## Materials and methods

### Experimental design

All mice were handled and sacrificed by our co-author, XW Mao, at Loma Linda University under AUP 8130028 in a previous study [31]. All data were obtained from cardiac tissue samples collected from that previous study. Cardiac tissue samples were obtained from mice in a previous study performed at Loma Linda University under a protocol approved by the Institutional Animal Care and Use Committee [31]. Mature (6 months old), female C57BL/6J mice were exposed to low-dose-rate γ-irradiation (Co-57), were subjected to HU, or received a simultaneous combination of HU and γ-irradiation (HU+Co-57) for 21 days as previously described [31]. Chronic low-dose rate irradiation was performed by housing mice for 21 days in cages placed on Co-57 plates (GPI; Stoughton, WI), as previously described [32–35]. Co-57 plates had an active area of 16.5 x 24 inches and a dose uniformity of ± 5%. Mice received a cumulative dose of 0.04 Gy at an average dose rate of 0.01 cGy/h. Dose calibration was performed with multiple thermoluminescent dosimeters per cage, as detailed in prior studies [32–35]. Mice were hind-limb unloaded as previously described [36,37]. In short, anti-orthostatic suspension was achieved by attaching a tail harness and suspending mice by the tail harness to elevate the hindquarters above the cage floor with a 30° head-down tilt. Mice remained in HU for the duration of 21 days. After completion of the 21 days of HU, γ-irradiation, or a simultaneous exposure to HU + γ-irradiation, all mice were removed from HU and the radiation source until the time of sacrifice. Mice not undergoing HU or γ-irradiation served as controls (CTL).

All mice were provided standard mice chow and water *ad libitum* and were housed on a 12:12 hour light:dark cycle. Mice were allowed normal ambulation after completion of the 21 day treatment period and were sacrificed 7 days, 1 month, 4 months, or 9 months later. Hearts were collected, either fixed in 4% paraformaldehyde or flash frozen in liquid nitrogen, and shipped to the University of Arkansas for Medical Sciences for analysis. Mice were monitored daily during HU, γ-irradiation, or the combined HU+ γ-irradiation treatment and every other day following completion of their 21 day treatment. While the study began with a total 6 animals per group, HU is not always tolerated well by animals, and a total of 4 mice were removed from the study due to significant decreases in body weight (weight loss greater than 15% within 2 weeks). Additional samples were depleted through biochemical analyses, which limited the n size for some of the analyses. Sample size for each experimental group is indicated in Figure legends.

### High-performance liquid chromatography

Markers of oxidative stress and methylation state were measured by high-performance liquid chromatography with electrochemical detection (HPLC-ECD), as previously described [38,39]. Briefly, 50 mg of frozen heart samples were homogenized in 500 μl of phosphate buffered saline solution (4°C). Following homogenization, samples were incubated (4°C; 30 min) in 10% metaphosphoric acid (100 μl tissue homogenate:150 μl acid) and then centrifuged at 18,000g (4°C; 15 min). Supernatant (20 μl) was injected into the HPLC reverse phase C_18_ Shisheido column (Phenomenex® Inc.) via ESA Autosampler (ESA Inc.). HPLC was performed to assess levels of reduced glutathione (GSH), oxidized glutathione (GSSG), methionine, S-adenosyl-L-methionine (SAM), and S-adenosyl-L-homocysteine (SAH) and expressed in nmol/mg total protein. Glutathione oxidation was expressed as a ratio of GSH:GSSG and methylation potential was expressed as a ratio of SAM:SAH.

### Immunoblot analysis

Immunoblot analysis was performed as previously described [40,41]. In brief, frozen heart samples were homogenized with a Potter-Elvehjem homogenizer in 1% Triton-X100 RIPA buffer containing a protease inhibitor cocktail (Sigma-Aldrich; 1:100) and a phosphatase inhibitor cocktail (Sigma-Aldrich; 1:100). Homogenates were centrifuged at 14,000 rpm for 10 min at 4° C and the supernatant removed for analysis. Protein concentration was determined with a BCA protein assay kit (Sigma-Aldrich), and 25 μg protein was loaded into Criterion TGX 4–20% gradient gels, electrophoresed, and transferred to PVDF membranes. After incubation with 1° and 2° antibodies diluted in 5% non-fat dried milk, membranes were developed using Immobilon™ Western Chemiluminescent HRP Substrate (Millipore) and CL-Xposure film (Thermo Scientific). Immunoblotting was performed to determine protein content of α-smooth muscle cell (SMC) actin (Abcam; 1:2,000), mast cell tryptase (Santa Cruz Biotechnology, Inc.; 1:25,000), caspase-3 (Santa Cruz Biotechnology, Inc.; 1:3,000), CD-2 (Santa Cruz Biotechnology, Inc.; 1:3,000), collagen-α1 type III (Santa Cruz Biotechnology, Inc.; 1:1,000), 4-hydroxynonenal (4-HNE) (gifted from Sharda Singh, Ph.D.; 1:10,000), manganese superoxide dismutase (MnSOD) (Santa Cruz Biotechnology, Inc.; 1:20,000), glutathione peroxidase (GPX 1/2) (Santa Cruz Biotechnology, Inc.; 1:500), catalase (Santa Cruz Biotechnology, Inc.; 1:1,000), Nrf2 (Santa Cruz Biotechnology, Inc.; 1:1,000), and GAPDH (Santa Cruz Biotechnology, Inc.; 1:20,000). Protein content was determined by densitometry, imaged with an AlphaImager® gel documentation system (ProteinSimple), and quantified with Image Studio Lite. In images where GAPDH showed double banding, both bands were included in the analysis. Protein content of all probed proteins was expressed relative to GAPDH.

### RNA isolation and real-time polymerase chain reaction

RNA isolation and rt-PCR was performed as previously detailed [40,42]. In short, RNA was isolated from frozen heart samples using Ultraspec™ RNA reagent (Biotecx). RNA quality and concentration were assessed spectrophotometrically using a NanoDrop® 2000. DNA was removed from the RNA with RQ-DNAse I (Promega), and cDNA was synthesized from the RNA using a High Capacity cDNA Reverse Transcription Kit (Life Technologies). Steady-state mRNA was assessed by real-time quantitative PCR (RT-qPCR). Gene expression was assessed using relative quantification (RQ) values obtained from TaqMan Gene Expression Assays™. RQ values were calculated as 2^-ΔΔCT^, where ΔΔCT = ΔCT_sample_− ΔCT_control_ and ΔCT represented the difference in cycle thresholds of a gene of interest and the housekeeping gene (18s rRNA).

### Fixation and histology

Mouse hearts were excised and placed in 4% paraformaldehyde (7.4 pH) for 24 hours, rinsed with PBS and dehydrated with ethanol. After dehydration, mouse hearts where embedded in paraffin wax and sectioned (5 μm) for histology, conducted as previously described [39,43].

To assess inflammatory cell infiltration, longitudinal heart sections were rehydrated and incubated with 0.5% Toluidine Blue in 0.5 N HCL for 3 days at room temperature, followed with a 10 minute wash in 0.7 N HCL, and were counterstained with Eosin (1:4). Mast cells were counted using an Axioskop transmitted light microscope (Carl Zeiss).

Cardiac remodeling was evaluated by collagen deposition and was determined using Sirius Red/Fast Green staining. Two series of sections were obtained from each heart, with the second series of histology samples sectioned approximately 50 μm deeper into the embedded mouse hearts, to provide a measure of collagen deposition throughout the heart. Heart sections were rehydrated and incubated with Picro Sirius Red supplemented with Fast Green. Sections were scanned using a ScanScope CS2 slide scanner, analyzed with ImageScope 12 (Leica Biosystems), and collagen deposition was determined as a percentage of the total tissue area, i.e. the relative area of collagen (Pico Sirius Red staining) to the total area of the tissue.

### Statistics

All values are means ± standard error of the mean (SEM). NCSS 8 (Kaysville, UT) was used to perform repeated measures analysis of variance (ANOVA) on collagen deposition in histology or one-way ANOVA for all other parameters, followed by Tukey-Kramer post-hoc tests of differences among individual experimental groups. Statistical significance was defined as p < 0.05. With 6 mice per group, we had 0.80 power to detect a difference of 1.7 SDs between an irradiated and sham group within a time point, based on a .05 significance level *t*-test conducted within an ANOVA framework. With 3 mice per group, we had 0.80 power to detect a difference of 3.3 SDs. Here, SD is the root mean square error from the ANOVA.

## Results

### Indications of cardiac oxidative stress and anti-oxidant enzyme expression

**Fig 1:**
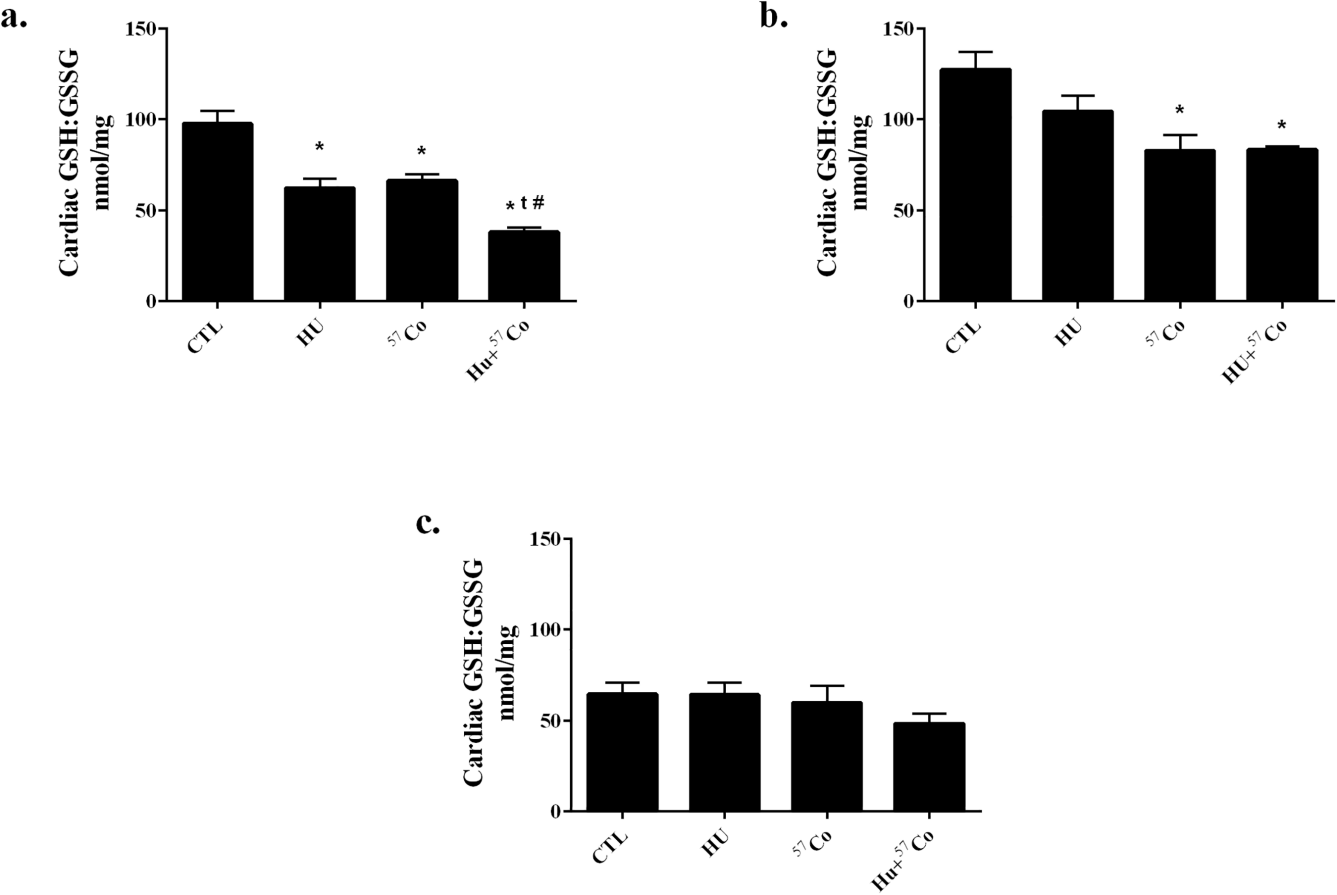
GSH:GSSG ratios in mouse heart following HU, γ irradiation or combined HU + γ irradiation. HPLC was utilized to determine GSH:GSSG ratios a.) 7 days, b.) 1 month, or c.) 9 months after a 21 day exposure to HU, γ irradiation (^57^Co: 0.01 cGy/h; 0.04 Gy total), or combined HU + γ irradiation. Sample sizes: CTL for all time points, n = 6; HU for 7 day time-point, n = 4; HU for 1 month time-point, n = 5; HU for 9 month time-point, n = 6; ^57^Co for 7 day and 9 month time-points, n = 5; ^57^Co for 1 month time-point, n = 6; HU+^57^Co for 7 day time-point, n = 6; HU+^57^Co for 1 and 9 month time-points, n = 5. Values are means ± SEM. * Significantly different than CTL, p < 0.05; t Significantly different than HU, p < 0.05; # Significantly different than ^57^Co, p < 0.05.

Cardiac tissue GSH (S1 Fig) and GSSG (S2 Fig) levels were assessed and GSH:GSSG ratios were calculated as an indication of oxidative stress (Fig 1). GSH:GSSG ratios were significantly decreased 7 days and 1 month after completion of HU or γ-irradiation. The combination of HU and γ-irradiation showed a further significant decrease in GSH:GSSG ratio than either treatment alone at the 7 day time point (Fig 1A), but not at 1 month (Fig 1B). Statistical analysis did not indicate significant differences in GSH:GSSG ratio in cardiac tissue collected at the 9 month time point (Fig 1C). Despite an early decrease in the GSH:GSSG ratio, no changes were observed in protein 4-HNE adducts as measured by immunoblot analysis (S3 Fig).

A small reduction in cardiac MnSOD expression was observed 9 months after low-dose rate irradiation, but no alterations were seen in MnSOD at 4 months, or GPX 1/2 at 4 or 9 months (S4 Fig). Furthermore, no statistical differences were observed in Nrf 2 or catalase protein content at 4 months or 9 months after treatment (S5 Fig).

### Cardiac methylation potential

**Fig 2:**
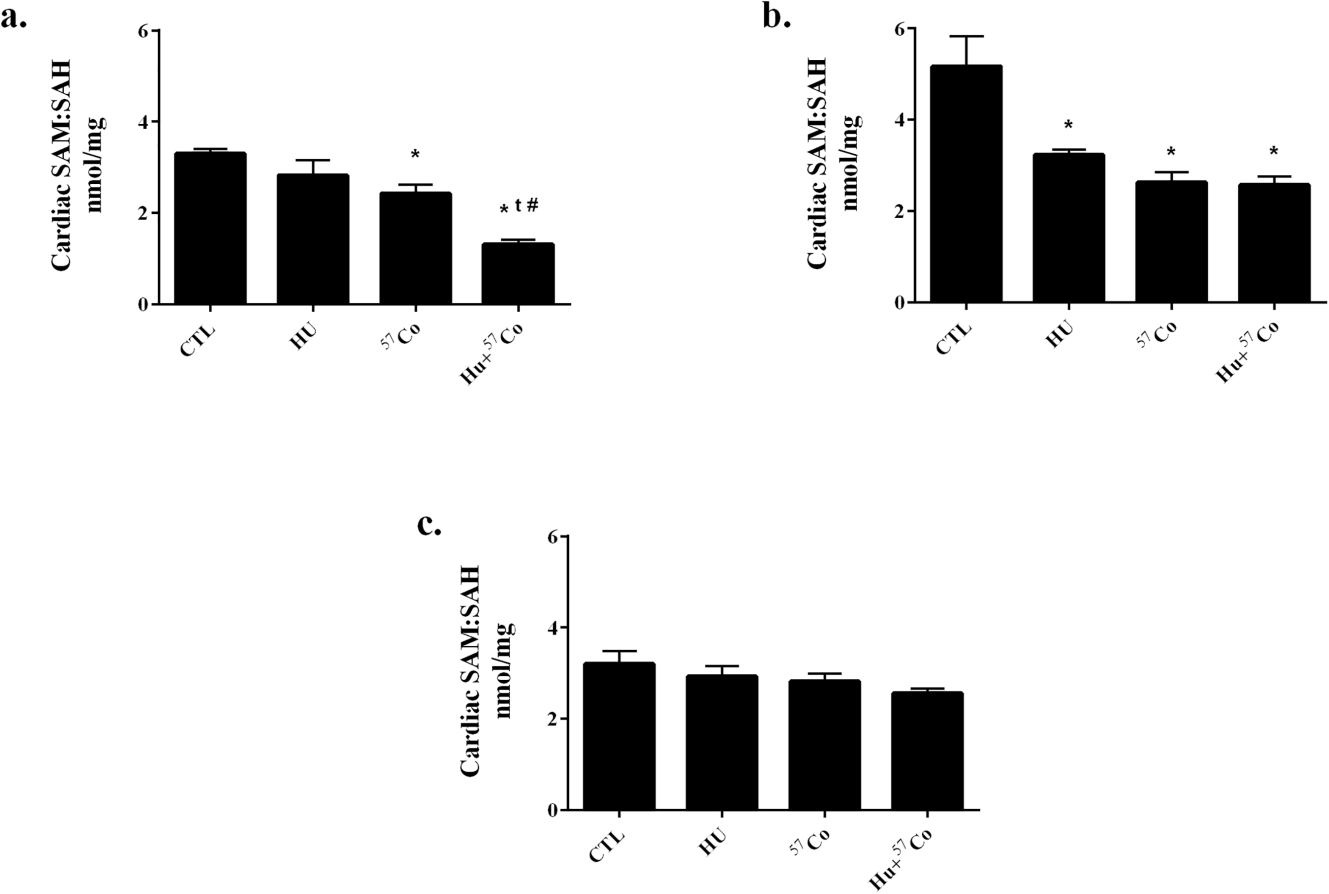
SAM:SAH ratios in mouse heart following HU, γ irradiation or combined HU + γ irradiation. HPLC was utilized to determine SAM:SAH ratios a.) 7 days, b.) 1 month, or c.) 9 months after a 21 day exposure to HU, γ irradiation (^57^Co: 0.01 cGy/h; 0.04 Gy total), or a combined HU + γ irradiation. Sample sizes: CTL for all time points, n = 6; HU for 7 day time-point, n = 4; HU for 1 month time-point, n = 5; HU for 9 month time-point, n = 6; ^57^Co for 7 day and 9 month time-points, n = 5; ^57^Co for 1 month time-point, n = 6; HU+^57^Co for 7 day time-point, n = 6; HU+^57^Co for 1 and 9 month time-points, n = 5. Values are means ± SEM. * Significantly different than CTL, p < 0.05; t Significantly different than HU, p < 0.05; # Significantly different than ^57^Co, p < 0.05; $ Significantly different than HU+^57^Co, p < 0.05.

A small reduction in SAM (S6 Fig) and increase in SAH (S7 Fig) levels in the heart as measured 7 days after completion of HU and low-dose rate γ-irradiation resulted in a significant reduction in the ratios of SAM:SAH (Fig 2A). The combination of HU and γ-irradiation showed a further reduction in SAM:SAH ratio compared to either HU or γ-irradiation alone (Fig 2A). After 1 month, the SAM:SAH ratio was significantly decreased in all groups compared to CTL, but the combined HU+ γ-irradiation group did not show an additional decrease (Fig 2B). There were no significant differences in SAM:SAH ratio in cardiac tissue collected after 9 months (Fig 2C), or in cardiac methionine content at any time point (S8 Fig).

### Cardiac collagen content

**Fig 3:**
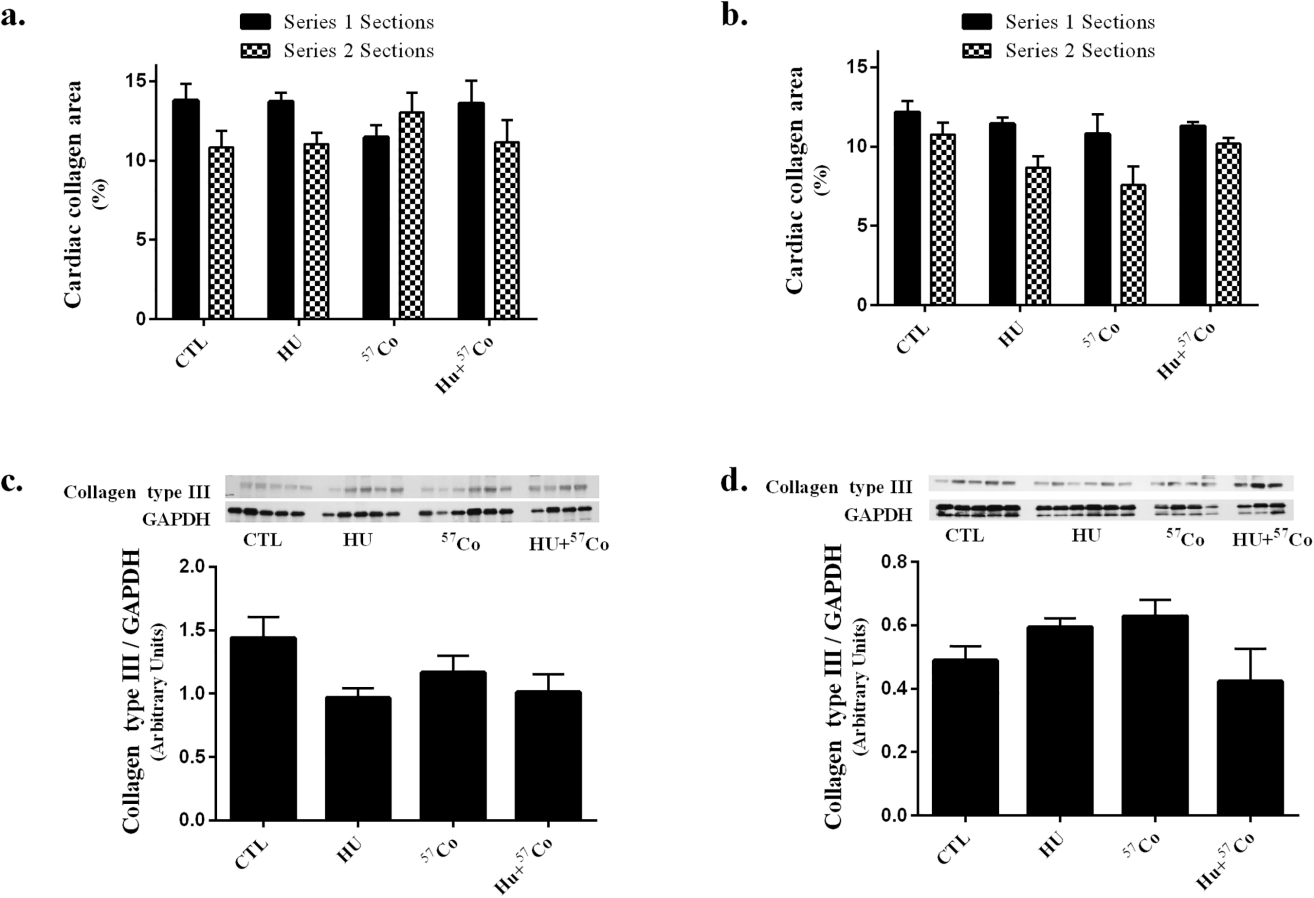
Collagen deposition in mouse heart following HU, γ irradiation or combined HU + γ irradiation. Sirius Red + Fast Green histological staining for total collagen a.) 4 months and b.) 9 months after treatment. Two series of sections were obtained from each heart, with the second series of histology samples sectioned approximately 50 μm deeper into the heart. Statistical analysis was performed by one-way ANOVA and repeated measures ANOVA. Histology sample sizes: CTL for 4 month time-point n = 4; CTL for 9 month time-point n = 5; HU for 4 month time-point n = 5, HU for 9 month time-point n = 6; ^57^Co for 4 month time-point n = 5; ^57^Co for 9 month time-point n = 5; HU+^57^Co for 4 month time-point n = 3, HU+^57^Co for 9 month time-point n = 5. Values are means ± SEM. Immunoblot analysis of collagen type III protein content c.) 4 months and d.) 9 months following HU, γ irradiation or combined HU + γ irradiation. All proteins were normalized to GAPDH. Immunoblotting sample sizes: CTL for 4 month time-point n = 4; CTL for 9 month time-point n = 5; HU for 4 month time-point n = 5, HU for 9 month time-point n = 6; ^57^Co for 4 month time-point n = 6; ^57^Co for 9 month time-point n = 4; HU+^57^Co for 4 month time-point n = 4, HU+^57^Co for 9 month time-point n = 3. Values are means ± SEM. Statistical analysis did not reveal any significant differences.

Since cardiac adverse remodeling typically occurs late after exposure to ionizing radiation, indicators of remodeling and inflammatory infiltration were examined at the observation time points of 4 and 9 months. Histological staining did not reveal significant group differences in total % collagen, in the first series of sections obtained from each heart, the second series, or in a repeated measures analysis of all sections (Fig 3A and 3B). Additionally, both immunoblot and RT-PCR analyses revealed no significant differences 4 or 9 months after treatment in collagen-α1 type III protein content (Fig 3C and 3D) or pro-collagen III relative mRNA (S9 Fig). Furthermore, immunoblot analysis of cardiac α-SMC actin did not reveal any significant differences at the 4 or 9 month time points (S10 Fig).

### Cardiac T-lymphocyte infiltration and mast cell numbers

**Fig 4:**
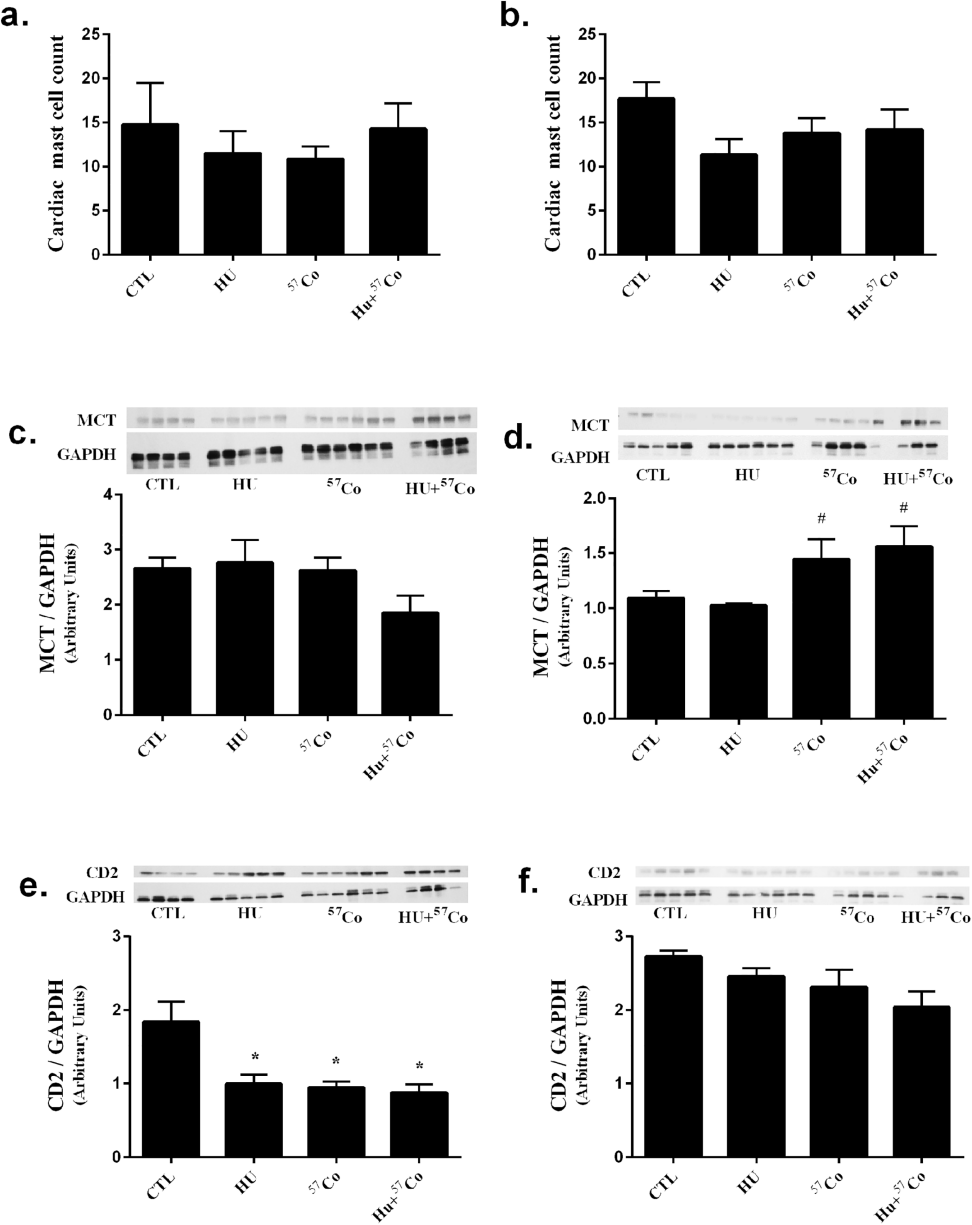
Mast cells and T-lymphocyte markers in mouse heart following HU, γ irradiation or combined HU + γ irradiation. Toluidine Blue histological staining for mast cell numbers a.) 4 months and b.) 9 months after HU, γ-irradiation (^57^Co: 0.01 cGy/h; 0.04 Gy total) or combined HU + γ-irradiation. Histology sample sizes: CTL for 4 month time-point, n = 4; CTL for 9 month time-point, n = 6; HU for both time-points, n = 6; ^57^Co for 4 month time-point, n = 6; ^57^Co 9 month time-point, n = 5; HU+^57^Co for 4 month time-point, n = 4; HU+^57^Co for month time-point, n = 5. Immunoblot analysis of mast cell tryptase (MCT) c.) 4 months or d.) 9 months after exposure. n = 3–6. Immunoblot analysis of CD-2 protein content e.) 4 months and f.) 9 months after treatment. Immunoblot sample sizes: CTL for 4 month time-point n = 4; CTL for 9 month time-point n = 5; HU for 4 month time-point n = 5, HU for 9 month time-point n = 6; ^57^Co for 4 month time-point n = 6; ^57^Co for 9 month time-point n = 5; HU+^57^Co for 4 month time-point n = 4, HU+^57^Co for 9 month time-point n = 3. All proteins are normalized to GAPDH. All values are means ± SEM. * Significantly different than CTL, p < 0.05; # Significantly different than HU, p < 0.05.

Mast cells were examined by histological staining with Toluidine Blue and by measuring mast cell tryptase content through immunoblot analysis. Histology did not reveal any significant differences in mast cell counts (Fig 4A and 4B); however, a small but significant increase in mast cell tryptase protein content was seen at 9 months after low-dose rate γ-radiation with and without HU (Fig 4C and 4D).

Cardiac CD-2 content was used as an indicator of T-lymphocyte infiltration. A significant decrease in CD-2 protein content was detected in all treatment groups compared to CTL in cardiac tissue collected 4 months after completion of HU, low-dose rate γ-irradiation or a combination of the two, but not in cardiac tissue collected at 9 months (Fig 4E and 4F).

## Discussion

The purpose of this study was to evaluate the impact of simulated microgravity and low-dose/dose-rate γ-irradiation on cardiac tissue. HU was used in this study as it is a widely accepted, terrestrial model of the mechanical unloading and fluid shifts that occur during exposure to microgravity [36,37]. HU was utilized in combination with exposure to low-dose rate γ-irradiation, in an effort to mimic the effects of a concurrent microgravity and radiation insult on the heart in a ground based model. More specifically, we assessed markers of oxidative stress, DNA methylation, inflammatory cell infiltration and remodeling in cardiac tissue obtained from 6 months old female C57BL/6J mice 7 days, 1 month and 9 months after completion of 21 days of HU, 21 days of exposure to low-dose rate γ-radiation, or 21 days HU concurrent with 21 day exposure to low-dose rate γ-radiation. This approach allowed us to study the effects of chronic low-dose rate radiation concurrent with HU. The animal model we have employed may also be relevant for low-dose/dose-rate γ-irradiation as may occur in certain populations on Earth, such as airline crew, miners, or radiological workers.

Glutathione, through its oxidation to glutathione disulfide, plays an important role in alleviating oxidative stress and has been used as an index of total tissue oxidative stress [44]. In the present study, GSH:GSSG ratios were decreased both 7 days and 1 month following exposure to HU or γ-irradiation. The data in our current study extend the finding of Sridharan et al., who used an animal model of high-dose local heart irradiation [39], by demonstrating that the GSH:GSSG ratio is sensitive to γ-radiation at a whole body, total low-dose of 0.04 Gy. Our data do not show a greater decrement in GSH:GSSG following 1 month of normal ambulation post-HU and no significant differences in GSH:GSSG ratios between groups were observed 9 months after treatment. The decrease in cardiac tissue GSH:GSSG 7 days and 1 month after irradiation combined with HU suggests that the heart may be more susceptible to oxidative insults. However, other indicators of oxidative stress such as the cardiac expression of Nrf2 and 4-HNE adducts did not significantly change at our measured endpoints, indicating that the hearts were not under severe oxidative stress at those times. Since the first post-treatment time point of analysis was 7 days, we cannot exclude that alterations in Nrf2 and/or 4-HNE may have occurred immediately after completion of HU or γ-irradiation. Moreover, 4-HNE alone is not an optimal indicator of lipid peroxidation. Therefore, future studies would benefit by including additional methods to measure lipid peroxidation, perhaps by malondialdehyde assay. These data suggest that a decrease in left ventricular GSH:GSSG ratios [39] results from an exposure to simulated microgravity or to low-dose/dose-rate γ-radiation, and that the combination of simulated microgravity and γ-irradiation produces a greater affect than either insult alone, measured up to one month after treatment. However, results from later time points suggest that a recovery occurs over the course of several months post-treatment.

Although large doses of γ-radiation have been shown to decrease SOD activity in rat hearts [45], low doses of γ radiation may increase SOD activity in some tissues; and SOD levels have been shown to increase in the heart following exposure to simulated microgravity, and further increase after reloading [46]. Proteomic analysis of heart tissue samples of Mayak workers that had been exposed to low doses of γ-radiation over prolonged periods of time showed a decrease in both Cu/ZnSOD and MnSOD protein levels in individuals who had received less than 0.1 Gy and individuals who had received more than 0.5 Gy [15]. On the other hand, Casciati et al. reported a decreased anti-oxidant response, including a decrease of Cu/ZnSOD protein content in hippocampus isolated 6 months after 2 Gy cranial x-ray irradiation of 10 day old mice, but not from mice irradiated at the lower dose of 0.1 Gy [47]. Proteomic analysis by Kempf et al. showed that chronic low-dose (0.3 Gy) γ-radiation is not associated with a decrease in anti-oxidant proteins, like SOD, but is associated with a decrease in lipid peroxidation in the hippocampus of Apo E^-/-^ mice [48]. The studies by Casciati et al. and Kempf et al. used doses of radiation that were several fold higher than the 0.04 Gy γ-radiation dose used in this study. However, a separate study by Mao et al., in the hippocampus collected from the same mice used in the current study, showed elevated 4-HNE levels 7 days and 9 months after HU or the combination of HU combined with chronic γ-irradiation [31]. Furthermore, Mao et al. also showed a decreased SOD activity 7 days after HU and 9 months after both HU and the combination of HU with γ-irradiation [31]. In contrast, our study of cardiac tissue did not elicit any differences in 4-HNE, GPX 1/2, and only a slight reduction in MnSOD protein content 9 months after γ-irradiation. Taken together, these data indicate that the alterations in oxidative stress and lipid peroxidation that occur following HU and low dose-rate γ-irradiation may be tissue specific.

Since the anti-oxidant glutathione may be modulated by the methionine cycle [24,25,49], we assessed cardiac methionine levels and SAM:SAH ratios. There were no changes in methionine levels at any time point. However, the SAM:SAH ratio was decreased at the 7 day and 1 month time point, with the combination group showing a further decrement than either HU or γ-irradiation alone. These alterations in the methionine cycle may impair DNA methylation, as suggested by Koturbash et al. [19], and may also contribute to reduced glutathione and potentially result in an increase in oxidative stress at these early time points.

High doses of radiation exposure are associated with structural remodeling of the heart, in part through increases in collagen content [1,50,51]. Proteomic and transcriptomic analyses suggest that structural remodeling after single, high-dose radiation exposure occurs through peroxisome proliferator-activated receptor alpha and transforming growth factor beta signaling [52]. Furthermore, structural remodeling of the heart may also occur after exposure to microgravity. For instance, Westby et al. reported a decreased left ventricular mass in subjects following exposure to simulated microgravity via head down tilt that persisted beyond the recovery of other cardiac mal-adaptations associated with simulated microgravity [53]. These results suggest that structural remodeling occurs following radiation exposure and simulated microgravity; however, our study, which used a low-dose, whole body γ-radiation, did not elicit a significant increase of collagen in any group. It is possible that the cumulative dose of 0.04 Gy radiation in the present experiment was too low to elicit the cardiac remodeling that occurs at higher doses [1,50,54]. While we used the same endpoints as previously used in animal models of high-dose radiation exposure, it is possible that we did not assess the appropriate parameters that indicate cardiac remodeling after HU or low-dose/dose-rate γ-irradiation.

We have previously shown that cardiac remodeling due to high-dose x-ray exposure is associated with increased cardiac mast cell numbers [50]. While the current study did not produce a change in mast cell numbers within the mouse heart after 4 months, we did observe a small but significant increase in mast cell tryptase protein content 9 months after low-dose γ-irradiation or a combination of HU + γ-irradiation by immunoblotting, which is likely a more sensitive measure to indicate the presence of mast cells compared to mast cell counts in histological analysis. Nonetheless, current data on collagen deposition and mast cell tryptase do not support the notion that extensive remodeling had occurred.

T-lymphocytes are an important modulator of the immune response, are highly sensitive to ionizing radiation [55], and among other inflammatory cells, are associated with cardiovascular disease [56]. Hence, we examined the common T-lymphocyte cell surface marker CD-2 as an indicator of cardiac T-lymphocyte infiltration. Previously, Patties et al. showed increased inflammatory markers 40 weeks after 8 and 16 Gy high-dose rate x-ray irradiation, but no significant differences after a low-dose 2 Gy irradiation [57]. Increased inflammation is associated with decreased cardiac function [58–60]. In contrast to Patties et al., we observed a significant decrease in T-lymphocyte CD-2 protein content 4 months after treatment. The chronic low-dose rate exposures in our study likely induce very different inflammatory responses compared to high-dose rate single dose irradiation. Alternatively, the decreased CD-2 protein content in our study may be reflective of a systemic decrease in T-lymphocytes, which may result in an impaired immune response after exposure to microgravity, low-dose radiation, or a combination of the two, which may recover over time. Space flight is associated with a compromised immune system [61,62], and data from Skylab and shuttle missions indicate a post-flight decrease in lymphocyte cell numbers [63–66]. Microgravity and low-dose rate γ-radiation may be responsible, in part, for the impaired immune function associated with spaceflight [63,65]. Future experiments should include complementary analyses, such as peripheral blood and cardiac T-lymphocyte cell counts.

In summary, exposure to 21 days of HU, low-dose γ-irradiation, or a combination HU + low-dose γ-irradiation decreased GSH:GSSG ratios, SAM:SAH ratios, and the inflammatory marker CD-2 protein content. These data suggest that exposure to both low-dose rate radiation and microgravity decreases methylation potential of the heart. The anti-oxidant protein MnSOD showed a small decrease 9 months after exposure to low-dose γ-radiation. However, this difference was not reflected in the other oxidative stress proteins we evaluated. Our analyses of collagen expression and α-SMC actin revealed no significant cardiac remodeling. It is possible that our study was limited by sample size and that we did not assess the appropriate parameters that indicate cardiac remodeling after HU or low-dose/dose-rate γ-irradiation. Further research is required to fully characterize the early and late effects of simulated microgravity combined with low-dose rate radiation on cardiac tissue.

## Supporting information

S1 Fig GSH concentration in mouse heart following HU, γ irradiation or combined HU + γ irradiation. HPLC was utilized to determine methionine concentration in mouse heart a.) 7 days, b.) 1 month, or c.) 9 month after a 21 day exposure to HU, γ irradiation (^57^Co: 0.01 cGy/h; 0.04 Gy total), or a combined HU + γ irradiation. Sample sizes: CTL for all time points, n = 6; HU for 7 day time-point, n = 4; HU for 1 month time-point, n = 5; HU for 9 month time-point, n = 6; ^57^Co for 7 day and 9 month time-points, n = 5; ^57^Co for 1 month time-point, n = 6; HU+^57^Co for 7 day time-point, n = 6; HU+^57^Co for 1 and 9 month time-points, n = 5. Values are means ± SEM. * Significantly different than CTL, p < 0.05.
(TIF)

S2 Fig GSSG concentration in mouse heart following HU, γ irradiation or combined HU + γ irradiation. HPLC was utilized to determine methionine concentration in mouse heart a.) 7 days, b.) 1 month, or c.) 9 month after a 21 day exposure to HU, γ irradiation (^57^Co: 0.01 cGy/h; 0.04 Gy total), or a combined HU + γ irradiation. Sample sizes: CTL for all time points, n = 6; HU for 7 day time-point, n = 4; HU for 1 month time-point, n = 5; HU for 9 month time-point, n = 6; ^57^Co for 7 day and 9 month time-points, n = 5; ^57^Co for 1 month time-point, n = 6; HU+^57^Co for 7 day time-point, n = 6; HU+^57^Co for 1 and 9 month time-points, n = 5. Values are means ± SEM. * Significantly different than CTL, p < 0.05; t Significantly different than HU, p < 0.05; # Significantly different than ^57^Co, p < 0.05.
(TIF)

S3 Fig 4-HNE protein content in mouse heart following HU, γ irradiation or combined HU + γ irradiation. Immunoblot analysis was used to determine 4-HNE protein content a.) 4 months and b.) 9 months after a 21 day exposure to HU, γ irradiation (^57^Co: 0.01 cGy/h; 0.04 Gy total), or a combined HU + γ irradiation. Sample sizes: CTL for 4 month time-point n = 3; CTL for 9 month time-point n = 5; HU for 4 month time-point n = 5, HU for 9 month time-point n = 6; ^57^Co for 4 month time-point n = 6; ^57^Co for 9 month time-point n = 5; HU+^57^Co for 4 month time-point n = 4, HU+^57^Co for 9 month time-point n = 3. Values are means ± SEM. Statistical analysis did not reveal any significant differences.
(TIF)

S4 Fig Antioxidant protein content in mouse heart following HU, γ irradiation or combined HU + γ irradiation. Immunoblot analysis was used to determine protein content of a.) MnSOD 4 months, b.) MnSOD 9 months c.) GPX1/2 4 months, and d.) GPX1/2 9 months following HU, γ irradiation (^57^Co: 0.01 cGy/h; 0.04 Gy total), or combined HU + γ irradiation. All proteins normalized to GAPDH. Immunoblot sample sizes: CTL for 4 month time-point n = 4; CTL for 9 month time-point n = 5; HU for 4 month time-point n = 5, HU for 9 month time-point n = 6; ^57^Co for 4 month time-point n = 6; ^57^Co for 9 month time-point n = 5; HU+^57^Co for 4 month time-point n = 4, HU+^57^Co for 9 month time-point n = 3. Values are means ± SEM. * Significantly different than CTL, p < 0.05.
(TIF)

S5 Fig Antioxidant protein content in mouse heart following HU, γ irradiation or combined HU + γ irradiation. Immunoblot analysis was used to determine protein content of a.) Nrf2 4 months, b.) Nrf2 9 months c.) Catalase 4 months, and d.) Catalase 9 months following HU, γ irradiation (^57^Co: 0.01 cGy/h; 0.04 Gy total), or combined HU + γ irradiation. All proteins normalized to GAPDH. Immunoblot sample sizes: CTL for 4 month time-point n = 4; CTL for 9 month time-point n = 5; HU for 4 month time-point n = 5, HU for 9 month time-point n = 6; ^57^Co for 4 month time-point n = 6; ^57^Co for 9 month time-point n = 5; HU+^57^Co for 4 month time-point n = 4, HU+^57^Co for 9 month time-point n = 3. Values are means ± SEM. * Significantly different than CTL, p < 0.05.
(TIF)

S6 Fig SAM concentration in mouse heart following HU, γ irradiation or combined HU + γ irradiation. HPLC was utilized to determine methionine concentration in mouse heart a.) 7 days, b.) 1 month, or c.) 9 month after a 21 day exposure to HU, γ irradiation (^57^Co: 0.01 cGy/h; 0.04 Gy total), or a combined HU + γ irradiation. Sample sizes: CTL for all time points, n = 6; HU for 7 day time-point, n = 4; HU for 1 month time-point, n = 5; HU for 9 month time-point, n = 6; ^57^Co for 7 day and 9 month time-points, n = 5; ^57^Co for 1 month time-point, n = 6; HU+^57^Co for 7 day time-point, n = 6; HU+^57^Co for 1 and 9 month time-points, n = 5. Values are means ± SEM. * Significantly different than CTL, p < 0.05.
(TIF)

S7 Fig SAH concentration in mouse heart following HU, γ irradiation or combined HU + γ irradiation. HPLC was utilized to determine methionine concentration in mouse heart a.) 7 days, b.) 1 month, or c.) 9 month after a 21 day exposure to HU, γ irradiation (^57^Co: 0.01 cGy/h; 0.04 Gy total), or a combined HU + γ irradiation. Sample sizes: CTL for all time points, n = 6; HU for 7 day time-point, n = 4; HU for 1 month time-point, n = 5; HU for 9 month time-point, n = 6; ^57^Co for 7 day and 9 month time-points, n = 5; ^57^Co for 1 month time-point, n = 6; HU+^57^Co for 7 day time-point, n = 6; HU+^57^Co for 1 and 9 month time-points, n = 5. Values are means ± SEM. * Significantly different than CTL, p < 0.05; t Significantly different than HU, p < 0.05; # Significantly different than ^57^Co, p < 0.05.
(TIF)

S8 Fig Methionine concentration in mouse heart following HU, γ irradiation or combined HU + γ irradiation. HPLC was utilized to determine methionine concentration in mouse heart a.) 7 days, b.) 1 month, or c.) 9 month after a 21 day exposure to HU, γ irradiation (^57^Co: 0.01 cGy/h; 0.04 Gy total), or a combined HU + γ irradiation. Sample sizes: CTL for all time points, n = 6; HU for 7 day time-point, n = 4; HU for 1 month time-point, n = 5; HU for 9 month time-point, n = 6; ^57^Co for 7 day and 9 month time-points, n = 5; ^57^Co for 1 month time-point, n = 6; HU+^57^Co for 7 day time-point, n = 6; HU+^57^Co for 1 and 9 month time-points, n = 5. Values are means ± SEM. Statistical analysis did not reveal any significant differences.
(TIF)

S9 Fig Pro-collagen III mRNA content in mouse heart following HU, γ irradiation or combined HU + γ irradiation relative to control 18s mRNA content. rt-PCR was used to determine pro-collagen III fold change 9 months after a 21 day exposure to HU, γ irradiation (^57^Co: 0.01 cGy/h; 0.04 Gy total), or a combined HU + γ irradiation. Sample sizes: all groups n = 4. Values are means ± SEM. Statistical analysis did not reveal any significant differences.
(TIF)

S10 Fig α-SMC actin protein content in mouse heart following HU, γ irradiation or combined HU + γ irradiation. Immunoblot analysis was used to determine protein content of a.) α-SMC actin 4 months and b.) α-SMC actin 9 months following HU, γ irradiation (^57^Co: 0.01 cGy/h; 0.04 Gy total), or combined HU + γ irradiation. All proteins normalized to GAPDH. Immunoblot sample sizes: CTL for 4 month time-point n = 4; CTL for 9 month time-point n = 5; HU for 4 month time-point n = 5, HU for 9 month time-point n = 6; ^57^Co for 4 month time-point n = 6; ^57^Co for 9 month time-point n = 5; HU+^57^Co for 4 month time-point n = 4, HU+^57^Co for 9 month time-point n = 3. Values are means ± SEM. * Significantly different than CTL, p < 0.05.
(TIF)

S1 Data The data used in the preparation of this manuscript. (XLSX)
